# Does the Length of Mini Dental Implants Affect Their Resistance to Failure by Overloading?

**DOI:** 10.3390/dj10070117

**Published:** 2022-07-01

**Authors:** Rafif Alshenaiber, Nick Silikas, Craig Barclay

**Affiliations:** 1 Division of Dentistry, Faculty of Biology, Medicine and Health, University of Manchester, Manchester M13 9PL, UK; nick.silikas@manchester.ac.uk; 2 Prosthetic Dental Sciences Department, College of Dentistry, Prince Sattam Bin Abdulaziz University, Al-Kharj 16278, Saudi Arabia; 3 Restorative Dentistry, University of Manchester Dental Hospital, Manchester M15 6FH, UK; craig.barclay@manchester.ac.uk

**Keywords:** compressive force, dental implant, static load

## Abstract

**Objective**: We aimed to evaluate the failure resistance of different lengths of mini dental implants from the same manufacturer, and to assess their failure following overloading. **Materials and Methods**: According to the ISO 14801, 15 mini dental implants 2.4 mm in diameter, with lengths of 8.5 mm, 10 mm, or 13 mm, were subjected to compression loading until failure using a universal testing machine. The mean load-to-failure values for each length of the mini dental implants were calculated and analysed using SPSS^®^, via one-way ANOVA (*p* < 0.05). **Results**: The mean load to failure for mini dental implants was 329 *N* (*SD* 6.23), 326 *N* (*SD* 5.95), and 325 *N* (*SD* 6.99) for the 13 mm, 10 mm, and 8.5 mm implants, respectively. A comparison of means showed no significant difference between the groups (*p* = 0.70). The tested mini dental implants exhibited bending failure modes below the first thread. **Conclusion:** Under high compressive loading testing, there was no effect of the length on the failure of the mini dental implants following overloading. Moreover, all tested mini dental implants with different lengths showed the same failure mode and distortion location.

## 1. Introduction

Residual ridge bone loss has been widely studied in the literature [1,2,3]; however, most of those studies involved investigating constructive methods rather than accepting the atrophic ridges as they are [4]. In atrophic ridges, the reduced width, height, and bone volume restrict conventional standard-length implant placement without further surgical intervention to optimise the implant position [1]. The recent trend in dentistry has been toward minimally invasive procedures. Therefore, choosing a narrower or shorter implant can provide less-invasive treatment [5,6]. Narrow implants, including mini dental implants (MDIs), can also be used to provide a minimally invasive dental implant treatment.

In edentulous atrophic ridges, conventional narrow dental implants may provide retention for overdentures. However, the abutment screw hole in a narrow conventional two-piece implant produces a fixture with a reduced cervical wall thickness. This design increases the risk of mechanical failure due to fracture or connection instability [7]. On the other hand, MDIs have a solid one-piece design that may reduce the risk of mechanical complications, favouring MDI overdentures as an alternative minimally invasive treatment option for atrophic ridges [8,9].

MDI overdentures may not always represent the first treatment option. However, the more conservative treatment option can be the first suitable option for some patients due to their social, medical, and financial status. Patients with two MDI overdentures reported high satisfaction levels, as their mastication and denture comfort were improved [10,11].

Today, several implant companies provide MDIs with the same original one-piece design but improved features to provide better outcomes for definitive treatment. Despite the reduced diameter, it is not uncommon to struggle to place the MDI within the residual ridge with anatomical hard and soft tissue boundaries—particularly for placement in severely atrophic ridges. Therefore, evaluating the residual ridges in terms of height and configuration is vital to tackle the prosthetic and surgical challenges during MDI placement in a residual atrophic ridge [1].

Short dental implants could negate the vertical augmentation of compromised residual ridge height [12]. They can also be used to avoid bone perforation or injury to vital structures within the residual ridges [13]. With the increasing popularity of prosthetic MDIs, most companies that supply MDIs manufacture different diameters and lengths, similar to conventional implants. Consequently, severely atrophic residual ridges with compromised bone width and height could be rehabilitated conservatively using short MDIs. However, short MDIs combine both reduced length and reduced diameter, which might compromise their mechanical and biological survival [14]. Nevertheless, implant length has been shown to have less impact on producing undesirable biomechanical stresses compared to implant diameter [15].

Although the predictability of MDIs is controversial because of their reduced diameter, many studies have reported high MDI survival rates [16,17,18]. However, most of the scientific literature on this topic used the IMTEC Sendax MDI system, which was discontinued and rebranded in 2010 as the 3M ESPE MDI, which was also discontinued six years later. Therefore, the available literature limits the generalisability of such MDI studies. Today, several manufacturers produce MDIs with improved manufacturing designs, as either implant fixtures, or attachment components similar to the one used in this study. In general, there is a gap in the literature regarding the long-term mechanical predictability of MDIs, let alone the effect of their length.

Failure of implants is multifactorial, and their length may contribute to failure. Therefore, investigating the in vitro mechanical failure of MDIs in relation to their length alone may help to understand how length contributes to the failure. This study compared extra-short MDIs, which can be used in severely resorbed ridges, to short and standard MDI lengths, with all other design factors being equal. The null hypothesis of this study states that there is no difference in the resistance to failure between the tested MDIs that have the same design features except for their length. The testing was carried out following the static testing part of the ISO 14801 dynamic loading test for endosseous dental implants. This testing protocol was designed to simulate the overloading of dental implants under worst-case conditions to compare the different designs and sizes of implants. However, the test was not used to predict the in vivo performance of dental implants [19].

## 2. Materials and Methods

**Figure 1:**
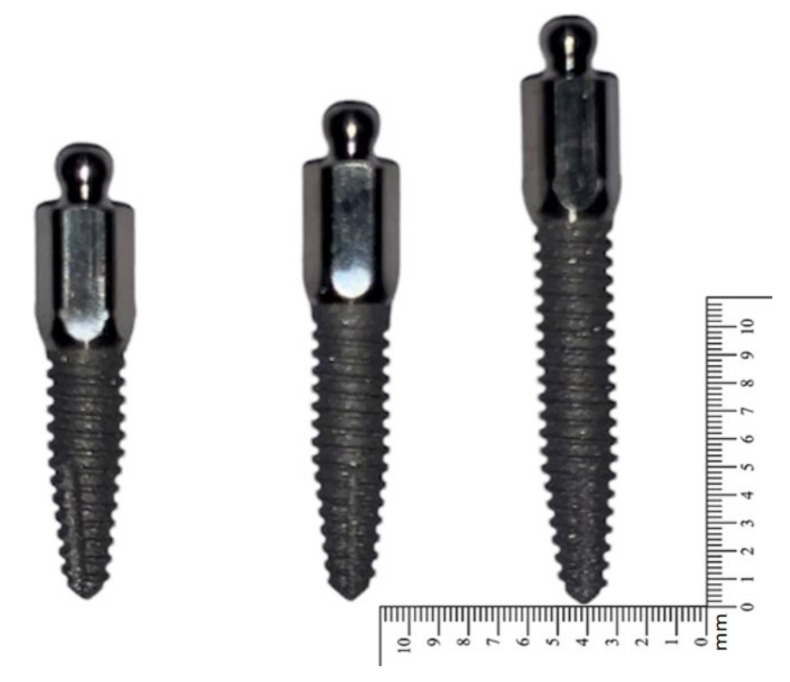
Straight ILZ MDIs from Southern Implants^®^ with a diameter of 2.4 mm. The length of the MDIs was 13 mm (**left**), 10 mm (**middle**), and 8.5 mm (**right**).

Fifteen straight ILZ MDIs (Southern Implants^®^, Irene, South Africa) of 2.4 mm width, with three different lengths of 8.5 mm, 10 mm, and 13 mm, were tested (Figure 1).

**Figure 2:**
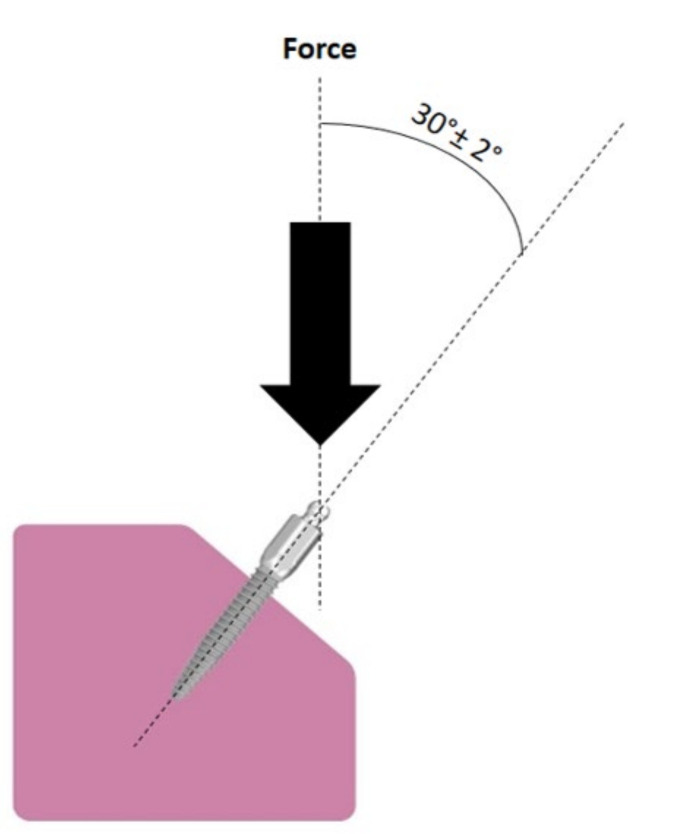
Scheme of implant sample set-up according to ISO 14801.

The samples of each length were prepared according to the British Standards ISO 14801: 2016 test specimen preparation guide [19]. The protocol indicates that the worst-case scenario of high loading should be mimicked during the test. This high-loading situation includes integrating the implant in Orthoresin blocks (Dentsply International, Inc., Milford, Del), which has a modulus of elasticity of more than 3 GPa. Each MDI was embedded into the resin using a dental surveyor (Austenal^®^ Surveyor, Skillbond Direct Ltd., Buckinghamshire, UK), exposing 3 mm of the implant fixture part to mimic marginal bone loss. The loading angle over the MDI was set at 30° to worsen the situation (Figure 2). The height of the abutment part was 6.6 mm, including a 1.8 mm ball attachment and a collar height of 4.8 mm, taking into account the 3 mm of bone resorption. Therefore, the coronal height of the samples tested was 9.6 mm, which acted as a lever arm.

**Figure 3:**
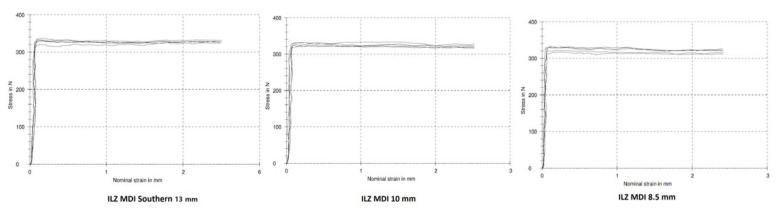
Stress–strain curves for mini dental implant samples of each length.

The compression load until failure was applied on the MDIs using a universal testing machine (Zwick Roell Z020, Herefordshire, UK) with a round-end loading rod, at a crosshead speed of 0.5 mm/min and loading cell of 500 *N*. The stress–strain curves and the load-to-failure values of all MDI samples were recorded using testXpert V 11.02 testing software (Figure 3).

The failure was determined as the movement of the loaded MDI of more than 0.5 mm or a sudden drop in the load–displacement curve. The mean load to failure (*N*) for each MDI length was calculated and analysed via one-way analysis of variance ANOVA using SPSS version 25, with a 95% confidence level (*p* < 0.05).

## 3. Results

**Figure 4:**
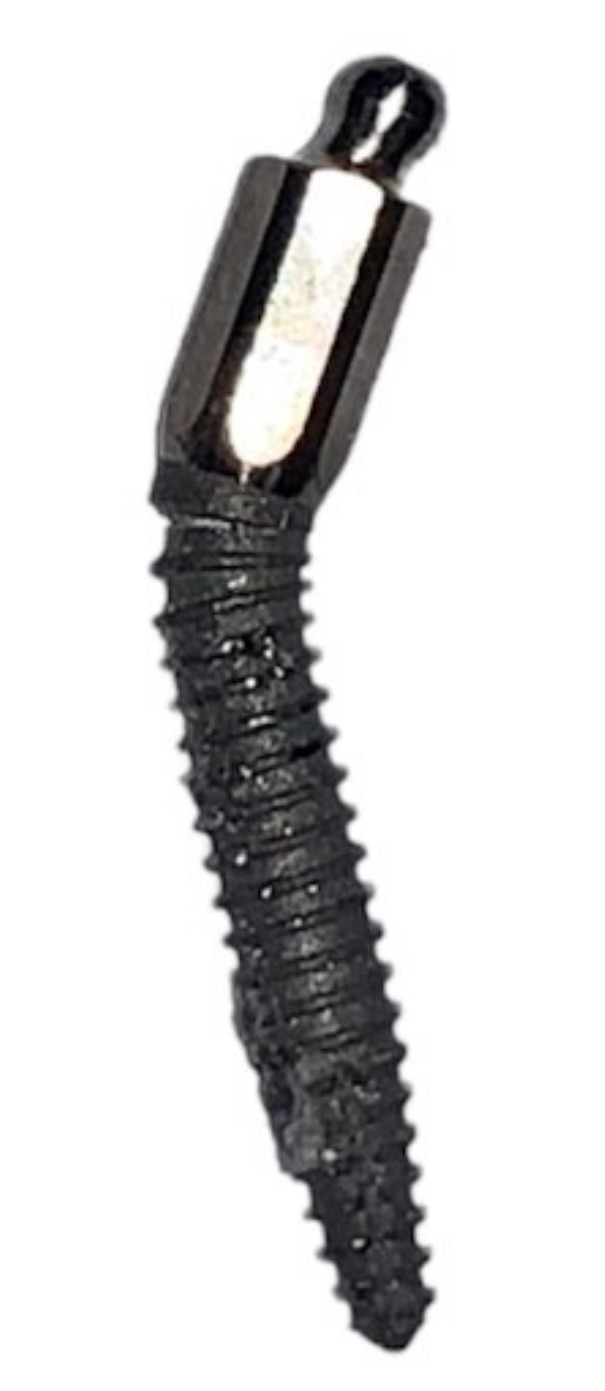
Retrieved mini dental implant sample from the resin block following testing, showing the bend under the first thread.

Following compression testing, the standard-length 13 mm MDI’s load-to-failure value was 329 *N* (*SD* 6.23). The load-to-failure values of short MDIs of 10 mm and extra-short MDIs of 8.5 mm were 326 *N* (*SD* 5.95) and 325 *N* (*SD* 6.99), respectively. A comparison of variance among the means was carried out using one-way ANOVA, and there were no significant differences between the tested MDI lengths (*p* = 0.70). Nevertheless, all MDI samples showed a similar failure mode following testing. Bending occurred at the fissure under the first thread, as shown in Figure 4.

## 4. Discussion

Some researchers refer to a dental implant with a length of 8 mm or less as a short implant [5]. However, others refer to a 10 mm implant as a short implant in their investigations [20]. This study refers to the 8.5 mm implant as an extra-short MDI, introduced to accommodate cases where the severe loss of residual ridge height is present. The association between implant location and length could present a dilemma when choosing an implant of appropriate length in an area of reduced bone—especially when combined with anatomical restrictions such as those posed by the mental nerve, lingual nerve, alveolar nerve, and maxillary sinus. A greater risk of failure was associated with the posterior placement of short implants [21]. Therefore, some have suggested that placing a short implant in an area where the risk of overloading is most significant may need to be avoided so as to reduce failure—particularly when combined with reduced implant diameter [14].

Extra-short MDIs for prosthetic rehabilitation might have a mechanical risk of failure, as they combine reduced length and width. Providentially, the results of this study show that such extra-short MDIs have similar resistance to failure compared to short and standard-length MDIs following overloading. Therefore, short MDIs could provide a more conservative treatment option in some cases. In addition, they may permit the placement of implants where the bone height is compromised, and where anatomical limitations restrict the use of standard-length MDIs. In residual atrophic ridges, a systematic review showed that conventional short implants provided fewer surgical complications and reduced the cost of treatment when compared to bone grafting followed by standard implants [12]. However, the clinical results on short MDIs are limited. One study investigated different implants with respect to their lengths and diameters, including MDIs. They found that long, narrow implants were subjected to higher stresses during placement via self-tapping [22]. This should be considered in future investigations of the placement of short and long MDIs in different bone types.

Another prospective cohort study that followed 21 patients with MDI overdentures for one year found that short MDIs had a failure rate of 38%, compared to a 3% failure rate of standard-length MDIs. Similar to the studies on conventional implants, all of the failures reported were related to biological loss of the implants, and not to mechanical failure [23]. With respect to the biological aspects of implant treatment, longer implants have a larger surface area, which improves the initial implant stability [24]. Hence, more short implants fail prior to mechanical loading [25].

Looking closely into the literature, we found two conflicting views about the effect of implant length on their survival. Some have found more biological complications associated with short implants, reducing their survival [26,27]. In contrast, reports from the year 2000 onward showed higher survival rates for shorter implants [20,28]. These contradictory results could be related to improved implant designs and surface characteristics, enhancing short implants’ success [29]. Consequently, one study showed that implant length was not associated with mechanical failure, but that design characteristics and manufacturing materials were correlated with mechanical implant failure [30].

Mechanical failure due to overload affected the area between the fixture and the collar of the MDI. The MDI has three major parts: abutment, collar, and fixture. The collar, or crest module, is located in the transitional area between the fixture and the abutment. The diameter of the collar is usually wider to provide a seal for the osteotomy. A wider collar enhances the implant surface area and reduces the stress at the crestal area of the bone [29]. The design of the MDI tested in this study has a wide collar, providing more bulk of the material in this area, which may improve the implant’s resistance to failure.

Findings by Pierrisnard et al. suggested that most stresses were concentrated on the cervical area of the first three threads of the fixture, regardless of the fixture length [31]. Failure mode in the form of bending following compressive testing mainly occurred at the first thread crest just below the collar, at the narrowest area of the MDI. Hence, this is consistent with other studies that conducted experimental and computer-simulated testing of MDIs [17,22]. The stress concentration around the neck area of the fixture was found to cause fracture or similar failure modes of narrow two-piece conventional implants [7]. MDIs offer a solid design at the neck; in contrast, narrow two-piece implants have thinner walls at the fixture’s cervical area, which house the abutment screw holes. Failure by bending at the abutment/implant connection could be catastrophic, as it may prevent the retrieval of the abutment, which precludes the implant’s restoration [32]. Therefore, MDIs as an alternative treatment may provide more resistance to mechanical failure by minimising the complications at the implant’s neck. However, more studies are needed to assess the differences between the two implant designs.

According to the manufacturer, the MDIs used in this study were designed to be placed in completely edentulous elderly patients. Narrow implants to rehabilitate the edentulous elderly are not new, as Huard et al. introduced the concept of geriatric slim implants in 2013 [33]. MDIs are usually placed in the anterior parts of ridges to support overdentures in atrophic ridges. Due to anatomical features and the common sequence of clinical tooth loss, more bone often remains in the anterior part of the residual ridges [34]. Moreover, elderly edentulous patients have recorded lower biting forces with dentures [35]. Fortunately, the anterior biting forces are less than the forces exerted in the posterior area [36]. Therefore, extra-short and short MDIs placed in the anterior residual ridges of elderly patients may not be subjected to much force when planned carefully.

However, the in vitro compressive load testing can be equated to the maximum biting force. The mean biting force of patients with two 1.8 mm MDI mandibular overdentures was about 191.5 *N* [37]. Moreover, the biting force was about 104.3 to 109.5 *N* measured in patients with four MDI overdentures of 1.8 mm and 2.1 mm MDI diameter [38,39]. Consequently, the load-to-failure values obtained from all MDI lengths were within the norms reported in the literature for maximum biting force in edentulous patients wearing MDI overdentures.

The ISO 14801 static loading test was carried out, providing data to understand the initial prediction of the performance of the implant [40]. It is essential to mention that static testing offers little clinical relevance compared to cyclic loading [41]. However, several studies have reported dental implant mechanical performance using only the static loading part of the ISO 14801 protocol. Several reasons were described that prevented the use of cyclic testing of the ISO 14801, including a large sample size, more extended testing periods, and increased cost [42,43].

Based on the limitations and results of this study, the data can provide a basis to predict the reliability of the MDIs’ function with respect to overloading, regardless of their length, providing that the test settings of this study simulated the worst loading conditions [19]. Such a testing methodology is helpful in understanding the MDI biomechanics in vivo, thereby optimising this treatment approach for patients. Consequently, due to the similar resistance to failure found using standard-length, short, and extra-short MDIs, the clinical use of shorter MDIs could be considered without compromising their mechanical strength.

## Data Availability

Data are available upon request from the corresponding author.
